# O042: Study on adopting the WHO 5-moment of hand hygiene for practices in traditional Chinese medicine (TCM) clinics

**DOI:** 10.1186/2047-2994-2-S1-O42

**Published:** 2013-06-20

**Authors:** P Ching

**Affiliations:** 1 Hong Kong Baptist Hospital, Hong Kong, Hong Kong

## Introduction

Hand hygiene (HH) is effective to prevent nosocomial infections in healthcare settings. However, the application of the WHO 5-moment for HH in TCM practices has not been reported. This study was performed to explore the feasibility of applying the WHO 5-moments for HH using alcohol-based handrub (ABHR) in TCM clinics. The objectives were to study when HH should be practiced, the feasibility of adopting the WHO 5-moments and the practicality of using ABHR in TCM practices.

## Methods

The clinic was visited to interview TCM practitioners and to understand the different practices performed and the extent of skin and blood and body fluid contacts during TCM practices. Direct observation of practices and videos on each procedure were performed to comprehend HH opportunities and assess the possibility of adopting the 5-moments. The frequency of HH action per hour and the need for personal protective equipment were estimated. Possible placement of AHR was identified.

## Results

The patient care and treatment practices in the TCM are different from western medicine and include: visit to a TCM practitioner, acupuncture with electric stimulation, moxibustion, cupping, and massage. TCM practitioner consultation, cupping and massage only involve skin contact and moment 1 and 4 are required. Acupuncture and moxibustion may cause limited blood exposure and moments 1, 3, 4 and 5 of HH are required after glove removal. Acupuncture, moxibustion and cupping require HH when applying related devices and upon removal. Practitioner consultations might need 3-4 HH actions per hour. Other treatments duration last 30 to 60 minutes and thus HH frequency is 1-2 times per hour. ABHR can be conveniently placed at couch and treatment trolley for use.

## Conclusion

The study demonstrates that TCM include 5 treatment practices that are different from western medicine. Limited blood exposures would occur during acupuncture and moxibustion while moments 1, 3, 4 & 5 are frequently recorded. The HH opportunities range from 2 to 4 per hour and are practicable. This preliminary study anticipated that HH using ABHR is doable in TCM clinic and WHO 5-moments for HH can be applied in the TCM practices. Tool kit and education for TCM practitioners will be developed. Further studies and pilot implementation will be conducted.

## Disclosure of interest

None declared.

